# Kinetic sampling shows the effect of medium composition on metabolic control in *Saccharomyces cerevisiae*

**DOI:** 10.1186/s12934-025-02884-w

**Published:** 2026-01-30

**Authors:** Marina de Leeuw, Lars Keld Nielsen

**Affiliations:** 1 The Novo Nordisk Foundation Center for Biosustainability, Technical University of Denmark, Kongens Lyngby, 2800 Denmark; 2 Department of Food Sciences, Institute of Postharvest and Food Sciences, Agricultural Research Organization - Volcani Institute, Rishon LeZion, 7505101 Israel; 3 Australian Institute for Bioengineering and Nanotechnology (AIBN), The University of Queensland, Brisbane, QLD 4072 Australia

## Abstract

Metabolic control analysis is used to understand regulation of metabolism and identify bottlenecks to be overcome in metabolic engineering for desired products. Its application has been hampered by the need for either parameterized models or carefully titrated experiments. In this study, we use thermodynamically feasible, sampled parameters to overcome this limitation. We use metabolic control analysis to explore central carbon metabolism of *Saccharomyces cerevisiae* growing in continuous culture under different nutrient limitations. Furthermore, we demonstrate shifts in flux control patterns in response to the different growth conditions and show how our results for specific reactions agree with the literature. Key advantages of the proposed framework include the incorporation of allosteric effectors, the use of omics data from a single steady-state time point and the computational efficiency; in all cases, 100 feasible models were sampled in less than 20 min on a laptop. The model and framework are freely available for researchers to use on their own data: https://github.com/biosustain/GRASP.git.

**Supplementary Information:**

The online version contains supplementary material available at 10.1186/s12934-025-02884-w.

## Introduction

*Saccharomyces cerevisiae* cells are popular cell-factories used to produce food, beverages, fuels, and chemicals [6, 15, 40]. There is a growing interest in the use of *Saccharomyces cerevisiae* cells to improve nutritional security by, for example, production of alternative proteins [23] and improvement of fermented food quality [16]. However, for the cells to compete with traditional production methods and offer a sustainable and cost-effective manufacturing process, they must perform optimally.

Advancements made in recent years in the fields of synthetic and computational biology supply us with both the tools and the knowledge required to design an efficient cell-factory. For example, we can now measure metabolite concentrations and metabolic fluxes in vivo, capturing the metabolic networks within a cell accurately [17]. We can also sequence genetic material and edit it to fit our production needs. And finally, we can use detailed computational models of cell metabolism to help us understand complex metabolic behaviors and suggest strategies for genome editing [8].

The metabolic behavior of the cell – and hence the optimal design – depends on its growth condition [42, 43]. For instance, Ye et al. [43] showed how the lipid production of the oleaginous yeast *Rhodosporidium toruloides* can be optimized by changing the carbon source and changing the ratio between carbon and nitrogen in the medium. Another well-known example is the Crabtree effect, in which *Saccharomyces cerevisiae* growing in aerobic conditions switches from respiration to fermentation in the presence of high glucose concentrations [26]. This dependence of cell metabolism on the growth medium should be considered when cell-factories are designed.

Kinetic metabolic models are ideal for capturing details of metabolism such as enzyme regulation [39], and hence ideal for analyzing metabolic control to identify strategies to optimize cell function under different conditions. Metabolic design models should capture the pathways leading to the desired product and preferably core metabolism that supply the requisite precursors. Thus, to model coumaric acid production, one would need to describe central carbon metabolism, aromatic amino acid metabolism, and the final synthetic steps from tyrosine or phenylalanine to coumaric acid. The parameterization of kinetic models is inherently challenging, but several strategies have been developed over the past decades to explore large metabolic networks [31, 35].

In *Saccharomyces cerevisiae*, kinetic metabolic models have been shown to accelerate the design–build–test–learn cycle in metabolic engineering of this widely used cell factory [33]. For instance, Narayanan et al. [22] employed a kinetic model to propose cell design strategies for the overproduction of *p*-coumaric acid, achieving a production increase of up to 32%. Similarly, Hu et al. [13] utilized a kinetic model to compare two distinct strains and identify key enzymes responsible for their metabolic differences. In both cases, the researchers successfully demonstrated how kinetic models can be used to study the metabolic consequences of genetic modifications.

In this study, we developed a kinetic model for the central carbon metabolism of *Saccharomyces cerevisiae* to investigate different metabolic control patterns due to changes in the cell growth medium. To develop our model, we used GRASP - a framework for thermodynamically consistent parameterization that enables the integration of allosteric mechanisms and the exploration of feasible samples within the solution space [28]. We sampled our model for nine different growth conditions using previously published omics data [10]. Then, we performed a metabolic control analysis (MCA) for each condition and investigated the shifts in the metabolic control patterns.

## Methods

### Model construction

Our *Saccharomyces cerevisiae* model was extracted from iMM904 model [21] and includes 82 reactions (including biomass drains, secretion and uptake drains) from central carbon metabolism. For each reaction, a kinetic mechanism was defined and, when known, competitive inhibitors, activators and allosteric effectors were stated (Table S1). The bounds of the Gibbs free energy for each reaction were calculated using eQuilibrator [24], while considering the physiological conditions in the cell compartments (pH 7.2 in cytosol and pH 7.5 in mitochondria [25]).

### Processing of omics data

Omics data (metabolomics and fluxomics) were obtained from Hackett et al. [10]. For each metabolite a minimum bound at 50% of the metabolite concentration and an upper bound at 150% of the metabolite concentration were set. The upper bound for metabolites found in mitochondria was set to 100 – fold higher, considering that the volume of the mitochondria is roughly 1% of the total cell volume [38] and allowing the extreme scenario in which all metabolite molecules are localized within the mitochondria. For metabolites in both the cytosol and the mitochondria, the lower bound was set to be 10^–15^ M for both. For unmeasured metabolites, the upper bound was set to an extremely high concentration of 10 M to and the lower bound was set to 10^–15^ M, to avoid unsupported constraints to the model. Finally, the concentrations of protons in the cytosol, in the mitochondria and outside the cell were set according to the pH in each compartment [25].

The fluxes for each reaction were calculated based on the data from Hackett et al. [10]. For the exchange fluxes of glucose, ethanol, acetate, glycerol, acetaldehyde and succinate, the difference between their concentration in the effluent and influent were used to calculate the flux into or out of the cell. The remaining fluxes were calculated based on the flux balance analysis of Hackett et al. [10]. Drains in and out of the cell (uptake and secretion) and biomass drains, were added when required to balance metabolites within our model.

### The GRASP framework

The constructed model and processed omics data were used as an input for the GRASP framework [18, 28, 29]. GRASP uses the data to sample the entire space of thermodynamically feasible elementary kinetic parameter sets [7, 30, 36]. This sampling technique allowed us to build a kinetic model without using literature kinetic parameters, which are not always available and depend on the conditions in which they were obtained [11, 37]. For each condition listed in Table 1, we ran GRASP until 100 thermodynamically feasible solutions were obtained. We repeated the process for selected conditions (cells growing in a glucose-limited medium) until 1000 samples were obtained. To establish linear stability, the Jacobian’s eigenvalues were calculated for each sampled model (a threshold was set to 1 × 10^− 5^).

The GRASP version used for this study was downloaded on May 10th, 2021, from the GRASP GitHub repository (https://github.com/biosustain/GRASP.git*).* We made minor modifications to the code and changed the temperature to 30 °C for the calculations of the Gibbs energies in two files: ‘computeGibbsFreeEnergyRanges.m’ and ‘sampleFluxesAndGibbsFreeEnergies.m’.

### Metabolic control analysis

The metabolic control coefficients of reactions in our model were calculated as explained earlier by Millard et al. [20]. In short, each flux control coefficient ($\:{C}_{{E}_{k}}^{{J}_{i}}$) quantifies the amount of control a reaction has on the metabolic network. It is defined as the percentage change in the flux *(J*_*i*_*)* per unit percentage change in the enzyme concentration *(E*_*k*_*)*.

$$\:{C}_{{E}_{k}}^{{J}_{i}}=\frac{\raisebox{1ex}{${\partial\:J}_{i}$}\!\left/\:\!\raisebox{-1ex}{${J}_{i}$}\right.}{\raisebox{1ex}{${\partial\:E}_{k}$}\!\left/\:\!\raisebox{-1ex}{${E}_{k}$}\right.}$$

**Fig. 1:**
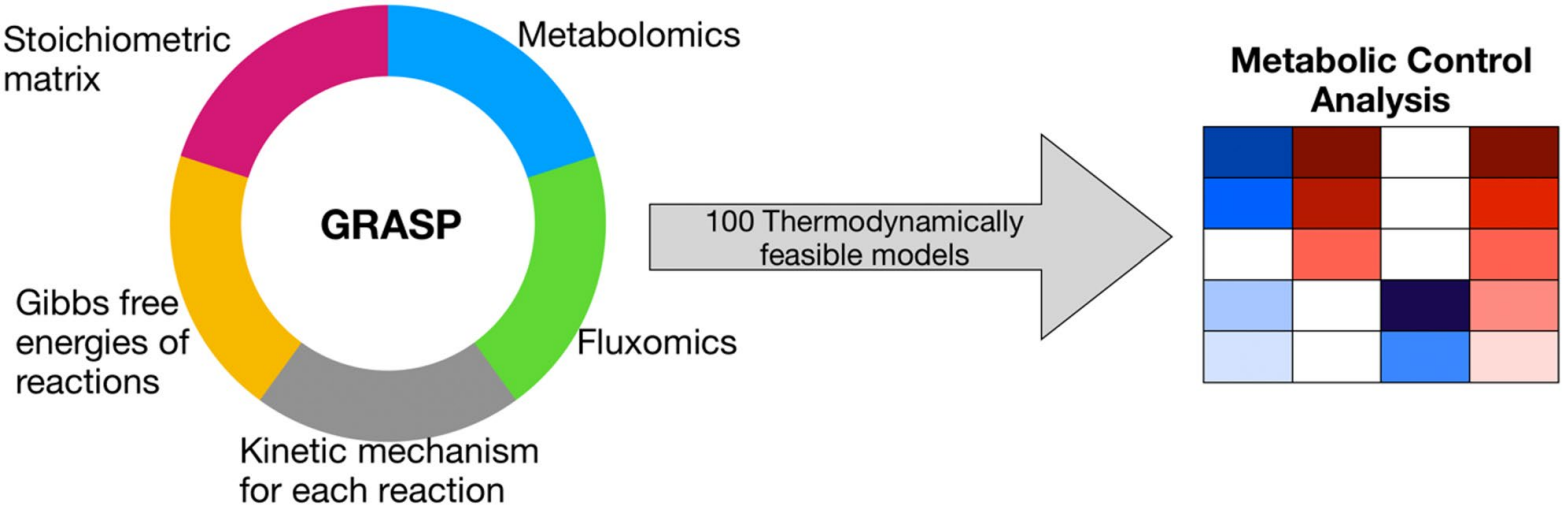
An illustration of the workflow

First, the flux control coefficient for each enzyme–flux pair ($\:{C}_{{E}_{k}}^{{J}{i}}$) was calculated for every sampled model. Then, for each growth condition, we calculated the median value over all sampled models for every enzyme–flux pair as illustrated in Fig. 1.

## Results and discussion

### Model assembly and sampling

Our kinetic model consists of 82 reactions, 52 metabolites and 3 compartments (cytosol, mitochondria and extracellular). The model includes reactions from the glycolytic pathway, the pentose phosphate pathway, ethanol metabolism and the TCA cycle (Fig. S2). Four of the model reactions are allosteric: Phosphofructokinase (PFK), Pyruvate kinase (PYK), Glycerol 3 Phosphate dehydrogenase (G3PD1ir) and Isocitrate dehydrogenase NAD (ICDHxm) [1, 2, 4, 34]. The complete model, including the kinetic mechanism defined for each reaction, is available in the GRASP GitHub repository (https://github.com/biosustain/GRASP.git*).*

For each condition, the model was sampled until 100 feasible models were obtained (i.e., 100 models x 9 growth conditions = 900 total models). The number of total runs and the average running time are shown in Table 1. As can be seen, half of the sampling sessions ended within 10 min on a MacBook Pro (2017 with a 2.3 GHz dual-core Intel core i5 processor) and all finished within 20 min. This renders GRASP a relatively fast sampling platform which can be easily used on personal computers.

**Table 1:** The time required to sample the metabolic models in this paper and the number of samples required to obtain at least 100 thermodynamically feasible models. For each sampling cycle, the algorithm was set to a minimum of 120 sampling attempts

Medium	Dilution rate [1/hr]	Run time [minutes: seconds]	Sampling attempts	Medium	Dilution rate [1/hr]	Run time [minutes: seconds]	Sampling attempts
Glucose-limited medium	0.30	4:43	132	Phosphorus-limited medium	0.11	10:30	343
	0.22	4:59	140	Nitrogen-limited medium	0.11	5:07	146
	0.16	3:59	120	Leucine-limited medium	0.11	10:52	343
	0.11	17:51	620	Uracil-limited medium	0.11	8:20	267
	0.05	3:55	120				

### Metabolic control analysis

Metabolic control analysis is a helpful tool to identify enzymes with a strong influence over the metabolic fluxes within a cell [18, 19]. To investigate whether the patterns of control change when cells are grown in different medium types, we calculated the control coefficients for all the enzymes in our model over all the fluxes as explained in Sect. 2.4. The process was repeated for all the assembled models listed in Table 1.

#### Influence of the glucose availability on metabolic control

After completing the metabolic control analysis, we compared the patterns for cells growing in five chemostats with varying dilution rates of the same glucose-limited medium [10]. We chose to investigate the growth of *Saccharomyces cerevisiae* in glucose-limited medium since there is literature on and understanding of the influence of glucose limitations on the metabolism of *Saccharomyces cerevisiae* [26].

**Fig. 2:**
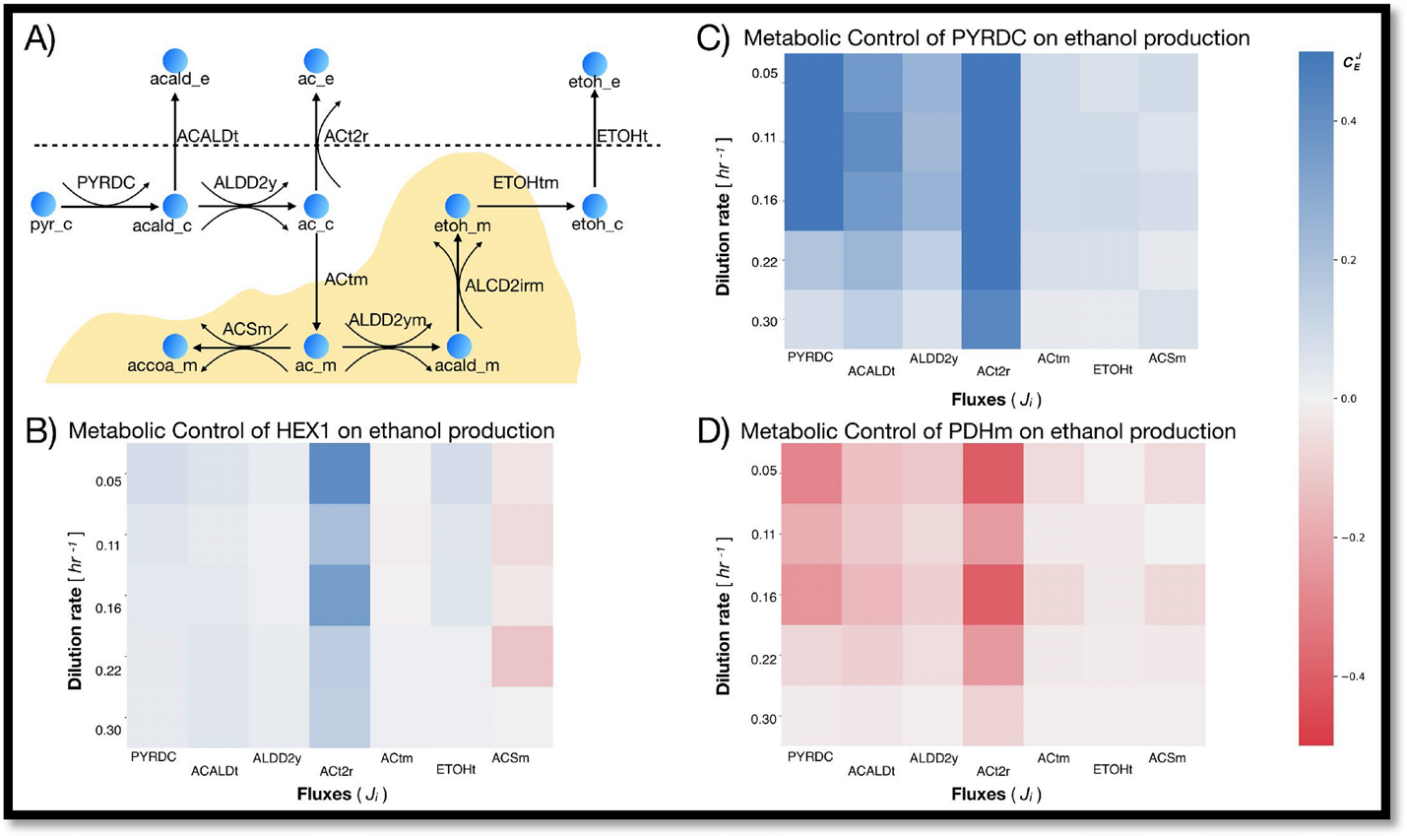
Heat-map of the metabolic control analysis for HEX1, PYRDC and PDHm on ethanol metabolism. Positive and negative median flux control coefficients ($\:{C}_{E}^{J}$) are represented by blue and red cells, respectively. **A** A scheme of the reactions leading to the production of ethanol in our model of *Saccharomyces cerevisiae. ***B**–**D** The metabolic control of hexokinase (HEX1), pyruvate decarboxylase (PYRDC) and pyruvate dehydrogenase (PDHm) on these fluxes. Plots (**B**–**D**) were made with seaborn [41] and Matplotlib [14]

Heat-maps showing the metabolic control analysis for the enzymes in our model (divided into four groups: glycolysis, pentose phosphate pathway, TCA cycle and ethanol production) can be viewed in the supplementary material (Figures S3-S7). Figure 2 shows the median control coefficients for hexokinase (HEX1), pyruvate decarboxylase (PYRDC) and pyruvate dehydrogenase (PDHm) on fluxes involved in ethanol production. As expected, HEX1 and PYRDC have a positive control over these fluxes (median values for 100 models) while the median control coefficients of PDHm are negative.

**Fig. 3:**
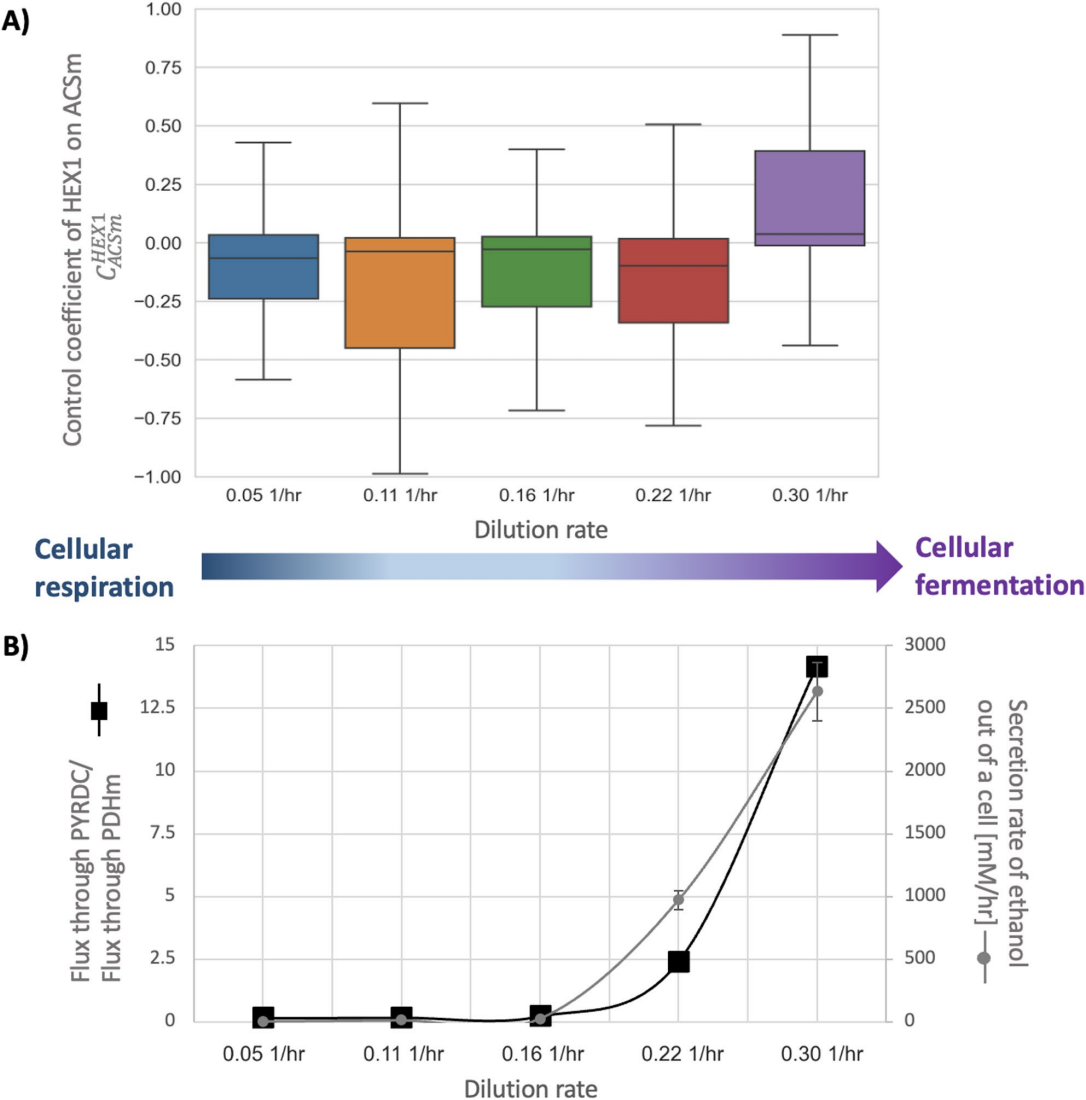
From cellular respiration to fermentation. **A** Flux control coefficients ($\:{C}_{E}^{J}$) for HEX1 on the flux through ACSm; Results for cells growing at five dilution rates in glucose-limited medium, 100 models for each condition. **B** Secretion rate of ethanol (grey circles) and ratio between the flux through PYRDC and PDHm (black squares) for cells growing in a glucose-limited medium (five dilution rates, data from Hackett et al. [10]). Plot (**A**) was made with seaborn [41] and Matplotlib [14]

The strength of the median control coefficients changes between the different dilution rates and gets generally weaker when more glucose is available. However, in contrast to the general trend, the median control coefficients for the dilution rate of 0.16 1/hr are in several cases stronger than those for 0.11 1/hr and 0.22 1/hr (e.g., HEX1 on the flux through ACt2r and PDHm on the flux through PYRDC). A possible explanation could be the transition of *Saccharomyces cerevisiae* cells from a respiratory state to a fermentative state when more glucose is available (Fig. 3B). The switch between the two states occurs somewhere between the 0.16 1/hr dilution rate and the 0.22 1/hr dilution rate. When the cells grow in a glucose-limited medium with a dilution rate of 0.16 1/hr, the flux through PYRDC is still lower than the flux of pyruvate into the mitochondria. However, when the dilution rate is raised to 0.22 1/hr the flux through PYRDC is more than twice the flux of pyruvate into the mitochondria.

As a result of the shift from respiration to fermentation, some control coefficients even change their sign. For example, HEX1 has a negative median control coefficient on the mitochondrial Acetyl-CoA synthase (ACSm) during respiration, and a positive median control coefficient during fermentation (Fig. 3A). This is unsurprising since both HEX1 and ACSm require ATP and compete for it. Hence, when glucose is limited in the medium, the conversion of glucose to g6p through HEX1 is prioritized, leaving fewer ATP molecules for ACSm. However, when there is sufficient glucose for fermentation (and enough acetate is produced from pyruvate) the median control coefficient for HEX1 on the flux through ACSm becomes positive.

After analyzing the control patterns for 100 sampled models, we repeated the process for 1000 sampled models (i.e., 1000 models x 5 growth conditions = 5,000 total models). We did not see a meaningful difference between the median values for each control coefficient when using the larger population of samples (Fig. S12: MCAs for cells growing in a glucose-limited medium with a dilution rate of 0.16 h^−1^, and Fig. S14(B): Median control coefficients for selected enzyme-flux pairs vs. number of sampled models). Additionally, we examined the distribution of control coefficients for selected enzyme-flux pairs, for example: the control of HEX1 on the fluxes through ETOHt, CO2tm and PYRDC (Fig. S13), we performed a Kolmogorov-Smirnov (K-S) test (Fig. S14A), and we examined the convergence of the median control coefficients (Figures S15-S16). Based on these results, we concluded that sampling 100 models was sufficient for our analyses.

#### Cells growing under varying conditions

**Fig 4.**
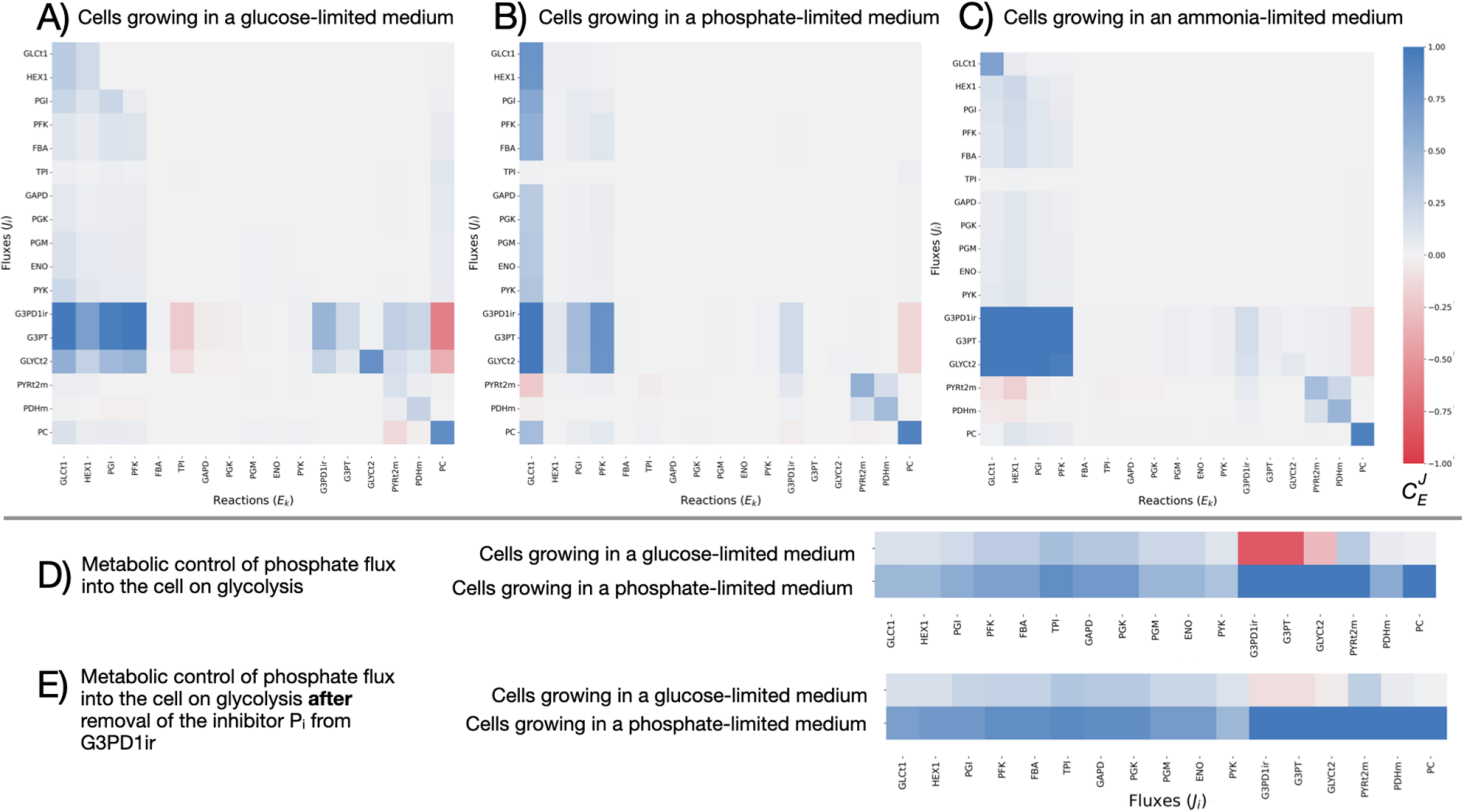
Metabolic control analysis for Saccharomyces cerevisiae growing under different conditions. Positive and negative median flux control coefficients ($\:{C}_{E}^{J}$ ) are represented by blue and red cells, respectively. **A** Cells growing in glucose-limited medium with a dilution rate of 0.11 1/hr; **B** Cells growing in phosphate-limited medium with a dilution rate of 0.11 1/hr; **C** Cells growing in ammonia-limited medium with a dilution rate of 0.11 1/hr; and **D** The metabolic control of the phosphate flux into our model on the glycolytic pathway for cells growing in the three different conditions. **E** The metabolic control of the phosphate flux into our model on the glycolytic pathway for cells growing in a glucose-limited medium after removing the inhibition of G3PD1ir by inorganic phosphate (P_i_). The plot was made with seaborn [41] and Matplotlib [14]

In this step, we analyzed the metabolic control patterns for cells growing in different types of medium. For simplicity, we show in Fig. 4 the analysis for cells growing only in three types of medium: (A) glucose-limited medium, (B) phosphate-limited medium and (C) ammonia-limited medium. The heat-maps for cells growing in other types of medium (i.e., Leucine-limited and Uracil-limited) and other metabolic pathways (ethanol metabolism, pentose phosphate pathway and TCA cycle) are available in the supplementary material file (Figures S4, S8-S11).

As can be seen in Fig. 4, we rarely observed changes in the signs of the calculated median control coefficient ($\:{C}_{E}^{J}$). Hence, reactions with a positive control on a flux have, in most cases, a positive control over the flux across all three media types, and the same can be said for reactions with negative control over fluxes. However, the strength of the median control coefficient varies. 

For example, the strength of the median control coefficient of hexokinase (HEX1), which uses ATP to convert glucose (glc__D) to glucose-6-phosphate (g6p), changes dramatically between the three growth conditions. When the cells grow in a phosphate-limited medium, the median values for hexokinase control coefficient are weaker (Fig. 4B) compared to its control coefficients for the other two growth conditions in Fig. 4 (light blue vs. dark blue shades in the heat map). A possible explanation is that when phosphate is scarce the control of HEX1 shifts to the import of glucose and inorganic phosphate to the cell through GLCt1 and a phosphate supply reaction (Fig. 4B and D, respectively).

**Fig. 5:**
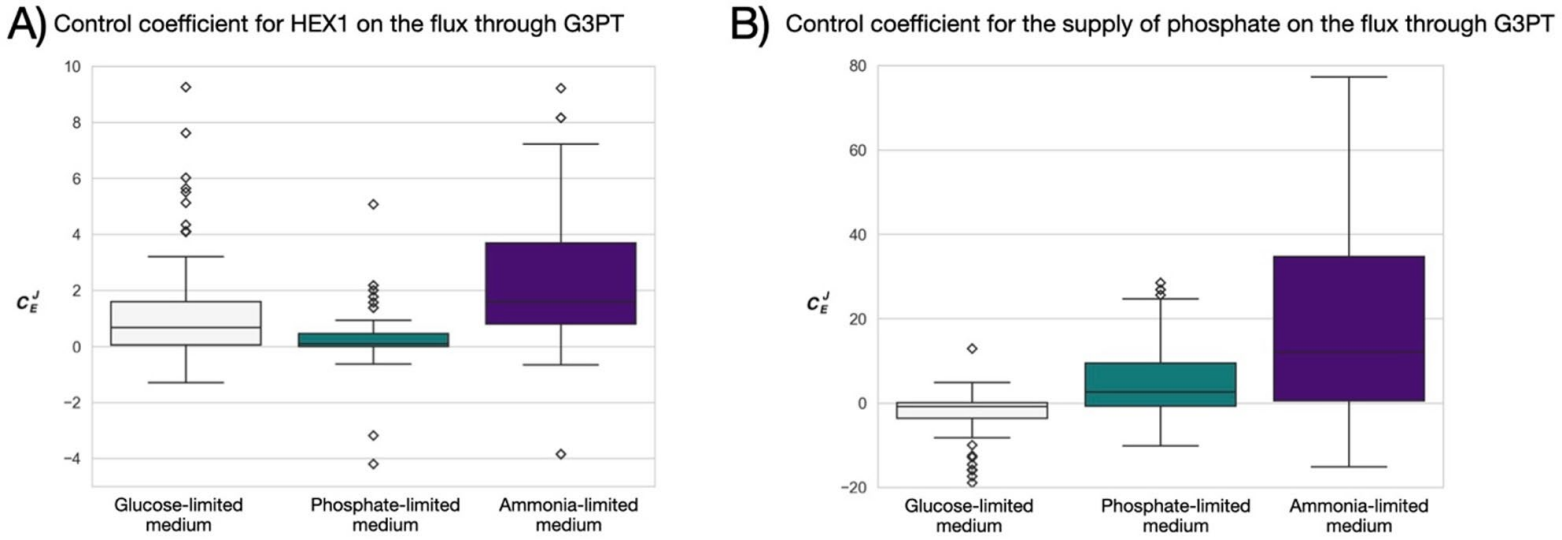
The influence of nutrient limitations (glucose-limited medium, phosphate-limited medium and ammonia-limited medium) on the distribution of (**A**) the control coefficient of HEX1 on the flux through G3PT, and (**B**) the control of the supply of inorganic phosphate on the flux through G3PT. The results presented are for 100 sampled models. Note the difference in the ranges of the y-axis in plots (**A**) and (**B**). The plot was made with seaborn [41] and Matplotlib [14]

An interesting change in the median control coefficient due to altering the growth conditions can be seen when examining the control of inorganic phosphate (P_i_) uptake drain on the glycolytic pathway (Fig. 4D). In this case, a negative control on glycerol production through glycerol-3-phosphatase (G3PT) when glucose is limited, changes into a positive control in the phosphate-limited medium (Fig. 5). A closer look at this reaction reveals G3PT converts glycerol-3-phosphate into glycerol, releasing inorganic phosphate into the cytosol. Furthermore, G3PD1ir is inhibited by inorganic phosphate [3, 32]. This explains why the control of phosphate uptake drain on the G3PT in our model is negative when glucose is scarce. However, when the cells grow in a phosphate-limited medium, the supply of inorganic phosphate has a strong positive control on the reactions leading to the formation of glycerol-3-phosphate (i.e., GLCt1, HEX1, PGI, FBA, and PFK - for which inorganic phosphate is a positive effector [27]). The ranges for the control coefficient for HEX1 and inorganic phosphate supply over G3PT can be examined more closely in Fig. 5.

These observations should be considered carefully as they depend not only on the metabolic reference data but also on the structure of the model. For example, the removal of the inhibition of G3PD1ir by inorganic phosphate, weakened its negative control over glycerol production in the glucose-limited media (Fig. 4E). In addition, the removal of the negative allosteric effector ATP from PFK lead to a stronger metabolic control of PFK over glycerol secretion (Fig. S17). Thus, a detailed mechanism, for each reaction is essential for the accuracy of the model, but mistakes in this mechanism and lack of precise knowledge may lead to inaccurate control patterns due to the conditional nature of MCAs [9]. It is expected that as more knowledge is gained regarding the mechanisms of each enzyme, kinetic models will become more accurate [5].

Nevertheless, our results clearly demonstrate that the growth medium significantly influences the delicate balance between the negative and positive effects of each enzyme on metabolic fluxes. Consequently, control patterns vary across different growth conditions, meaning a cell optimized for producing a specific metabolite in one condition may not do so efficiently in another. The kinetic model and sampling framework used in this study can help investigate changes in metabolic regulation and identify growth condition-specific targets for metabolic engineering, as part of the cell-factory design process.

## Conclusions

In this study, we demonstrated that it is possible to use GRASP, a framework for thermodynamically consistent parameterization and sampling, to investigate single steady-state data points and mine important information about their metabolic regulation. We applied GRASP to analyze an existing data set obtained for *Saccharomyces cerevisiae* growing in different growth conditions, under several nutrient limitations. Subsequently, we examined the metabolic control patterns for each condition separately and compared the regulation mechanisms between conditions.

The sampling process of each model was relatively fast and supplied important insights into the regulation patterns of the cell under different conditions. We observed shifts in the metabolic regulations between the studied conditions and showed how the control patterns are influenced by the structure of the model in addition to the environment in which the cell grows. These observations highlight the importance of considering the growth conditions of the cells when identifying bottlenecks (and enzymes with a positive control) as part of a cell-design process. We conclude that kinetic models and sampling platforms such as GRASP are essential tools for this type of analysis.

## Supplementary Information

Supplementary Material 1.

## Data Availability

The model and framework are freely available for researchers to use on their own data: https:/github.com/biosustain/GRASP.git.
