# Supplementary material for: Kinetic sampling shows the effect of medium composition on metabolic control in Saccharomyces cerevisiae
Source: Microb Cell Fact. 2026 Jan 30;25:76. doi: 10.1186/s12934-025-02884-w (PMC13019717; doi:10.1186/s12934-025-02884-w)
Supplement: Supplementary file 1 — Supplementary Material 1. [file 12934_2025_2884_MOESM1_ESM.pdf]

Supplementary Materials

Kinetic sampling shows the effect of medium composition on metabolic control in *Saccharomyces cerevisiae*

Marina de Leeuw and Lars Keld Nielsen

Title	Page
**Table S1:** Kinetic mechanism, inhibitors, activators, allosteric effectors and number of subunits for each allosteric reaction.	1-5
**Figure S2:** Visualization of the metabolic pathways included in the model.	6
**Metabolic control analysis heat-maps for nine different conditions, 100 sampled models for each condition**	
**Figure S3:** Metabolic control analysis heat-maps for cells growing in a glucose-limited medium with a dilution rate of 0.05 1/hr, 100 sampled models.	7
**Figure S4:** Metabolic control analysis heat-maps for cells growing in a glucose-limited medium with a dilution rate of 0.11 1/hr, 100 sampled models.	8
**Figure S5:** Metabolic control analysis heat-maps for cells growing in a glucose-limited medium with a dilution rate of 0.16 1/hr, 100 sampled models.	8
**Figure S6:** Metabolic control analysis heat-maps for cells growing in a glucose-limited medium with a dilution rate of 0.22 1/hr, 100 sampled models.	9
**Figure S7:** Metabolic control analysis heat-maps for cells growing in a glucose-limited medium with a dilution rate of 0.30 1/hr, 100 sampled models.	9
**Figure S8:** Metabolic control analysis heat-maps for cells growing in a phosphate-limited medium with a dilution rate of 0.11 1/hr, 100 sampled models.	10
**Figure S9:** Metabolic control analysis heat-maps for cells growing in a nitrogen-limited medium with a dilution rate of 0.11 1/hr, 100 sampled models.	10
**Figure S10:** Metabolic control analysis heat-maps for cells growing in an uracil-limited medium with a dilution rate of 0.11 1/hr, 100 sampled models.	11
**Figure S11:** Metabolic control analysis heat-maps for cells growing in a leucine-limited medium with a dilution rate of 0.11 1/hr, 100 sampled models.	11
**The Effect of Sample Size on the Performance of GRASP**	
**Figure S12:** Metabolic control analysis heat-maps for cells growing in a glucose-limited medium with a dilution rate of 0.16 1/hr. Positive values of C_E^J are indicated in blue, negative values are indicated in red. A) 100 sampled models, B) 1000 sampled models.	12
**Figure S13:** Distribution of the metabolic control coefficients for HEX1 on ethanol transport, carbon dioxide transport, and on the flux through PYRDC for cells growing in five different dilution rates of a glucose-limited medium. Blue: The first 100 sampled models out of 1000, Grey: All the 1000 sampled models.	13
**Figure S14:** (A) Kernel Density Estimation Plot showing the K-S test results (p-values) for comparison of metabolic control coefficient distributions between ensembles of 50, 75, 125 and 1000 models versus 100 models. (B) Median metabolic control coefficients for selected PRYDC-flux pairs.	14
**Figure S15:** Relative changes in median control coefficients vs. number of sampled models.	15
**Figure S16:** Normalized median control coefficients vs. number of sampled models.	15
**Allosteric effectors in our model**	
**Figure S17:** Kernel Density Estimation Plot showing the influence of ATP as an allosteric effector of PFK on the control of PFK and HEX1 over the secretion of glycerol.	16
**References**	17-18

Table S1: Kinetic mechanism, inhibitors, activators, allosteric effectors and number of subunits for each allosteric reaction. Note: the number of subunits is only indicated for allosteric enzymes. In most cases, the enzyme properties were obtained from [Schomburg et al. 2002; King et al. 2016; UniProt Consortium, T. (2018); Wang et al. 2021].

reaction ID	Enzyme Name	EC Number	kinetic mechanism	substrates	products	inhibitors	negative effectors	positive effectors	allosteric (Yes-1)	catalytic subunits	Additional References
ACONTm	Aconitate hydratase	4.2.1.3	UniUni	cit	icit h2o					1	
ACSm	Acetyl CoA synthetase	6.2.1.1	orderedTerTer	ac coa atp	ppi amp accoa					1	
ACt2r	Acetate reversible transport via proton symport	-	UnUni	ac_e	ac_c					1	
Actm	Acetate transport mitochondrial	-	UniUni	ac	ac					1	
ADK1m	Adenylate kinase	2.7.4.3	randomBiUni	amp atp	2adp					1	
AKGDam	Oxoglutarate dehydrogenase	1.2.4.2	orderedBiBi	akg lpam	sdhlam co2					1	
AKGDbm	Oxoglutarate dehydrogenase dihydrolipoamide S succinyltransferase	2.3.1.61	orderedBiBi	sdhlam coa	dhlam succoa					1 (multimer which forms 1 catalytic subunit)	
ALCD2irm	Alcohol dehydrogenase	1.1.1.1	orderedBiBi	acald nadh	etoh nad					1	
ALDD2y	Aldehyde dehydrogenase (acetaldehyde, NADP)	1.2.1.5	orderedBiBi	nadp acald	ac nadph					1	Bostian & Betts 1978
ALDD2ym	Aldehyde dehydrogenase acetylaldehyde NADP mitochondrial	1.2.1.5	orderedBiBi	nadp acald	ac nadph					1	Bostian & Betts 1978

ATPtm_H	ADPATP transporter mitochondrial	-	orderedBiBi	adp_c atp_m	adp_m atp_c					1	
CO2tm	CO2 transport diffusion mitochondrial	-	UniUni	co2_m	co2_c					1	
CSm	Citrate synthase	2.3.3.16	orderedBiBi	oaa accoa	coa cit					1 (homodimer, the monomer is inactive)	
ENO	Enolase	4.2.1.11	UniUni	2pg	pep					2	
ETOHt	Ethanol reversible transport	-	UniUni	etoh_e	etoh_c					1	
ETOHtm	Ethanol transport to mitochondria diffusion	-	UniUni	etoh_c	etoh_m					1	
FBA	Fructose-bisphosphate aldolase	4.1.2.13	orderedUniBi	fdp	dhap g3p					2	
FBA3	Sedoheptulose 1,7-bisphosphate D-glyceraldehyde-3-phosphate-lyase		orderedUniBi	s17bp	dhap e4p					1	
FUMm	Fumarase mitochondrial	4.2.1.2	UniUni	fum	mal__L					4	
G3PD1ir	Glycerol 3 phosphate dehydrogenase NAD	1.1.1.8	randomBiBiCompInhibitS1S2	nadh dhap	glyc3p nad	nad+ pi	atp adp		1	1	Cai et al. 1996
G3PT	Glycerol-3-phosphatase	3.1.3.2	orderedUniBi	glyc3p	glyc pi					1	
G6PDH2r	Glucose 6-phosphate dehydrogenase	1.1.1.49	orderedBiBi	g6p nadp	6pgl nadph					1	

GAPD	Glyceraldehyde-3-phosphate dehydrogenase	1.2.1.12	orderedTerBi	nad pi g3p	nadh 13dpg					4	McAlister and Holland 1985
GCC2cm	Glycine cleavage complex lipoamide mitochondrial	1.8.1.4	orderedBiBi	dhlam nad	nadh lpam					1	
GLCt1	Glucose transport uniport	-	UniUni	glc__D	glc__D					1	
GLYCt2	Glycerol transport via symport	-	UniUni	glyc_c	glyc_e					1	
GND	Phosphogluconate dehydrogenase	1.1.1.44	orderedBiTer	6pgc nadp	ru5p__D nadph co2					2	He et al. 2007
HEX1	Hexokinase (D-glucose:ATP)	2.7.1.1	orderedBiBiCompInhibS1	atp glc__D	g6p adp	adp		(cit_c is not in the model)		1	Hynne et al. 2001; Mulcahy et al. 2002; Gao and Leary 2003
ICDHxm	Isocitrate dehydrogenase NAD	1.1.1.41	orderedBiTer	icit nad	nadh akg co2			amp_m	1	4	Lin & McAlister-Henn 2003
MDHm	Malate dehydrogenase mitochondrial	1.1.1.37	orderedBiBi	nad mal__L	oaa nadh					2	
ME1m	Malic enzyme NAD mitochondrial	1.1.1.38	orderedBiTer	nad mal__L	pyr nadh co2					1	
PC	Pyruvate carboxylase	6.4.1.1	orderedBiTer	pyr atp	pi adp oaa					4	
PDHm	Pyruvate dehydrogenase	1.2.1.104	orderedTerTer	pyr coa nad	accoa nadh co2					1	
PFK	Phosphofructokinase	2.7.1.11	orderedBiBi	f6p atp	adp fdp		atp, (cit_c not in the model)	adp pi (cit_c and amp_c not in the model)	1	4	Przybylski et al. 1985
PFK_3	Phosphofructokinase (s7p)	2.7.1.11	orderedBiTer	s7p atp	adp s17bp h+					1	

PGI	Glucose-6-phosphate isomerase	5.3.1.9	UniUni	g6p	f6p					2	
PGK	Phosphoglycerate kinase	2.7.2.3	orderedBiBiCompInhibS1	3pg atp	adp 13dpg	adp (amp_c not in the model)				1	Rojas-Pirela et al. 2020
PGL	6-phosphogluconolactonase	3.1.1.31	UniUni	6pgl	6pgc					1	
PGM	Phosphoglycerate mutase	5.4.2.11	UniUni	2pg	3pg					4	
PYK	Pyruvate kinase	2.7.1.40	orderedBiBi	pep adp	atp pyr			fdp	1	4	Xu et al. 2012; Jurica et al. 1998
PYRDC	Pyruvate decarboxylase	4.1.1.1	orderedUniBiCompInhib	pyr	acald co2	pi				4	van Urk 1989
PYRt2m	Pyruvate mitochondrial transport via proton symport	-	UnUni	pyr_c	pyr_m					1	
RPE	Ribulose 5-phosphate 3-epimerase	5.1.3.1	UniUni	ru5p__D	xu5p__D					2	
RPI	Ribose-5-phosphate isomerase	5.3.1.6	UniUni	r5p	ru5p__D					1	
SUCCt2r	Succinate transport via proton symport	-	UniUni	succ_c	succ_e					1	
SUCCtm	Succinate transport mitochondrial	-	orderedBiBi	succ_c pi_m	succ_m pi_c					1	
SUCD2_u6m	Succinate dehydrogenase ubiquinone 6 mitochondrial	1.3.5.1	orderedBiBi	q6 succ	fum q6h2					1	

SUCOASm	Succinate CoA ligase ADP forming	6.2.1.5	orderedTerTer	succ coa atp	adp pi succoa					1 (one heterodimer)	
TKT1	Transketolase	2.2.1.1	pingPongBiBi	xu5p__D r5p	g3p s7p					2	Sevostyanova 2009, Solovjeva 2019
TKT2	Transketolase	2.2.1.1	pingPongBiBi	xu5p__D e4p	g3p f6p					2	Sevostyanova 2009, Solovjeva 2019
TPI	Triose-phosphate isomerase	5.3.1.1	UniUni	dhap	g3p					2	
**Exchange Reactions**			Mass Action								
**Metabolite drains**			Mass Action								

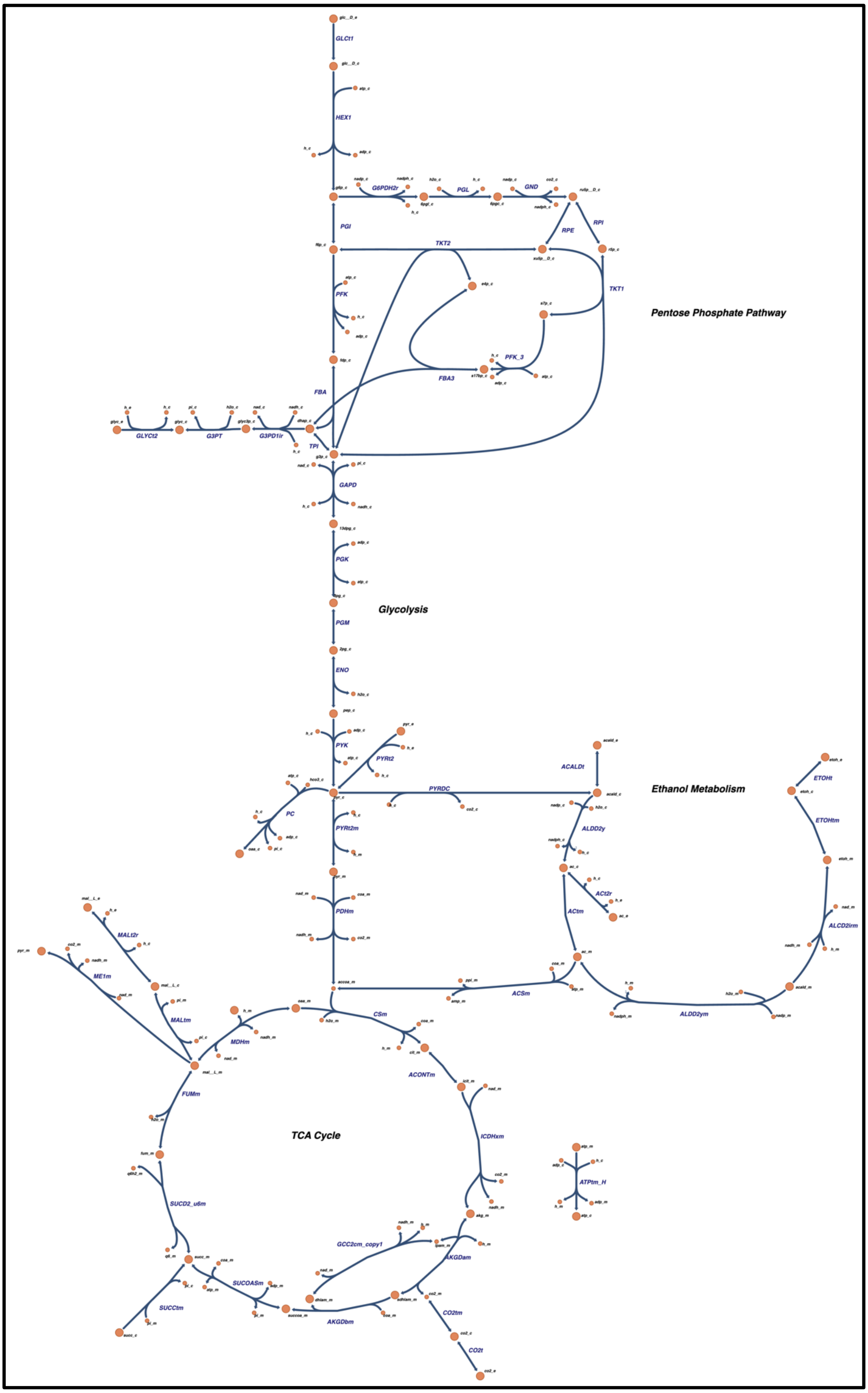

Figure S2: Visualization of the metabolic pathways included in the model. The figure was made using Escher web (King et al. 2015).

Metabolic control analysis heat-maps for nine different conditions, 100 sampled models for each condition

Plots S3-S11 were made with seaborn (Waskom 2021) and Matplotlib (Hunter 2007).

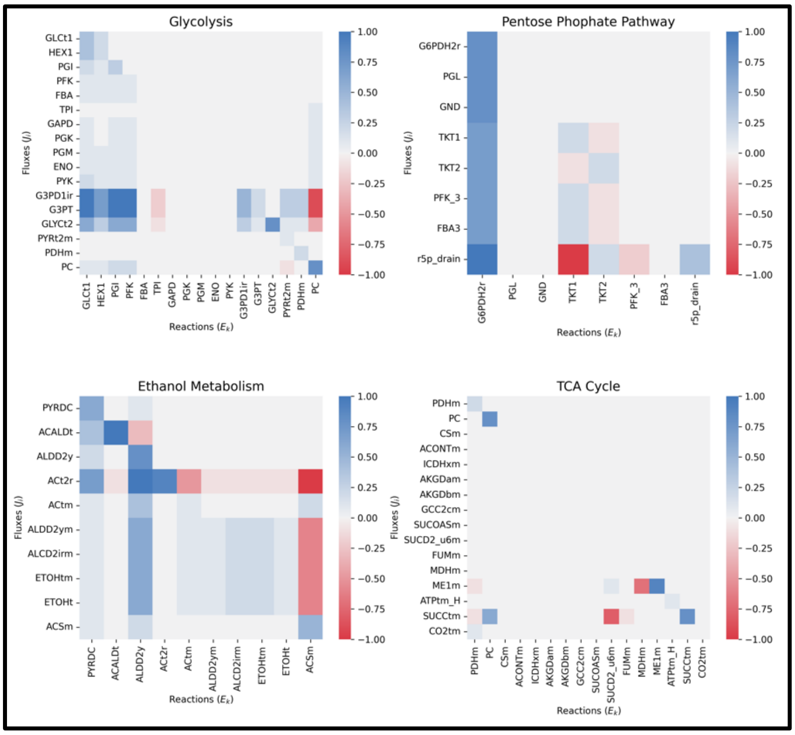

Figure S3: Metabolic control analysis heat-maps for cells growing in a glucose-limited medium with a dilution rate of 0.05 1/hr, 100 sampled models. Positive values of C_E^J are indicated in blue, negative values are indicated in red.

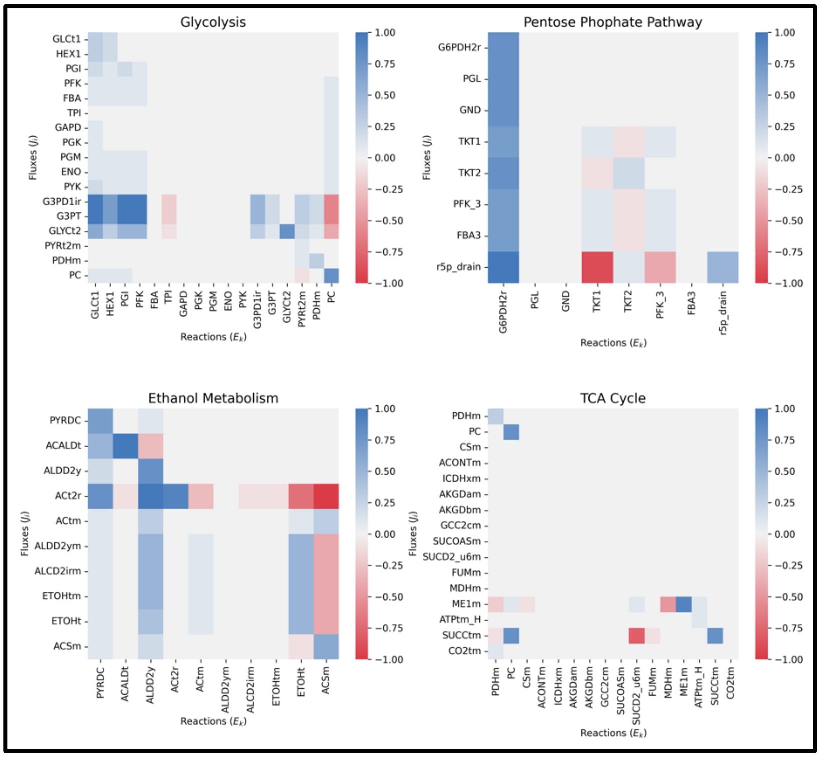

Figure S4: Metabolic control analysis heat-maps for cells growing in a glucose-limited medium with a dilution rate of 0.11 1/hr, 100 sampled models. Positive values of C_E^J are indicated in blue, negative values are indicated in red.

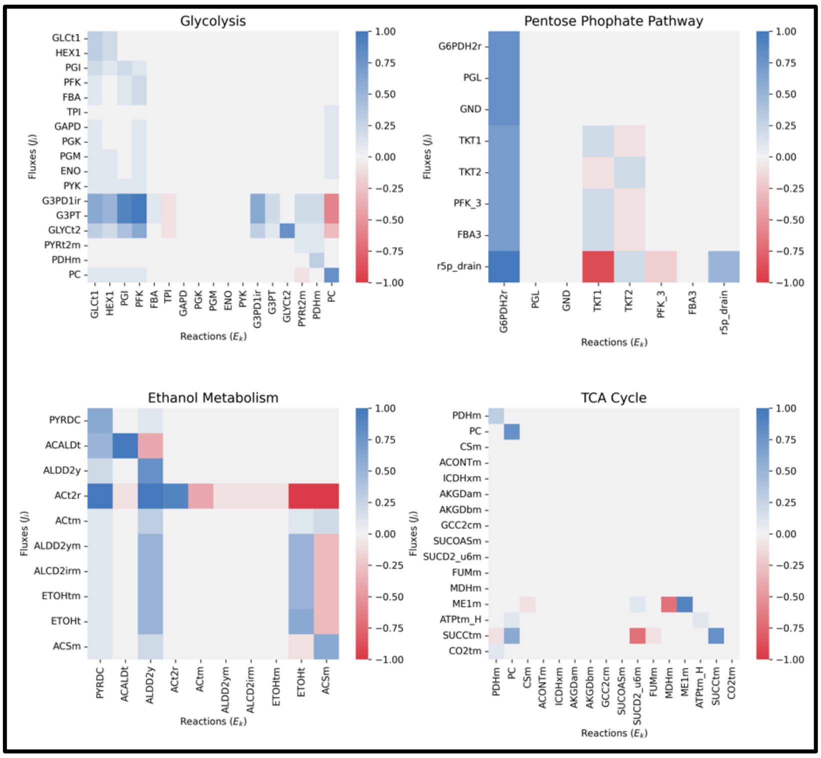

Figure S5: Metabolic control analysis heat-maps for cells growing in a glucose-limited medium with a dilution rate of 0.16 1/hr, 100 sampled models. Positive values of C_E^J are indicated in blue, negative values are indicated in red.

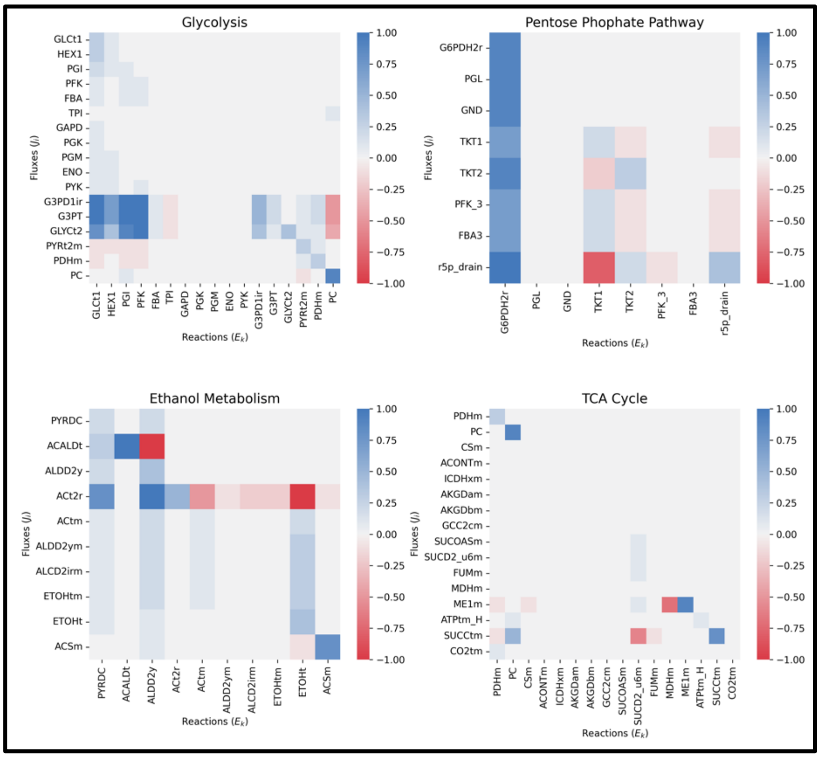

Figure S6: Metabolic control analysis heat-maps for cells growing in a glucose-limited medium with a dilution rate of 0.22 1/hr, 100 sampled models. Positive values of C_E^J are indicated in blue, negative values are indicated in red.

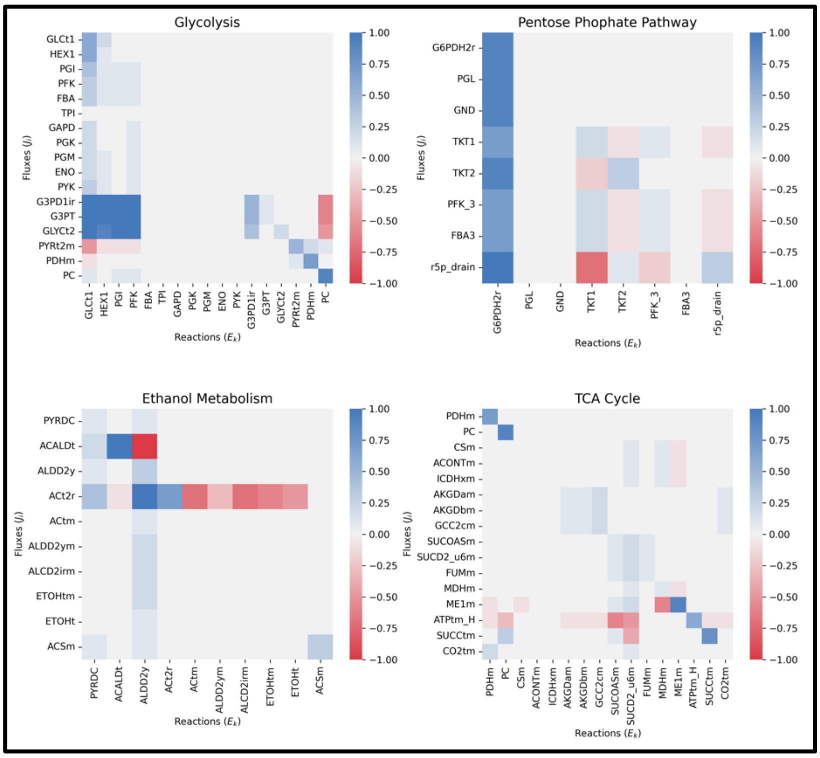

Figure S7: Metabolic control analysis heat-maps for cells growing in a glucose-limited medium with a dilution rate of 0.30 1/hr, 100 sampled models. Positive values of C_E^J are indicated in blue, negative values are indicated in red.

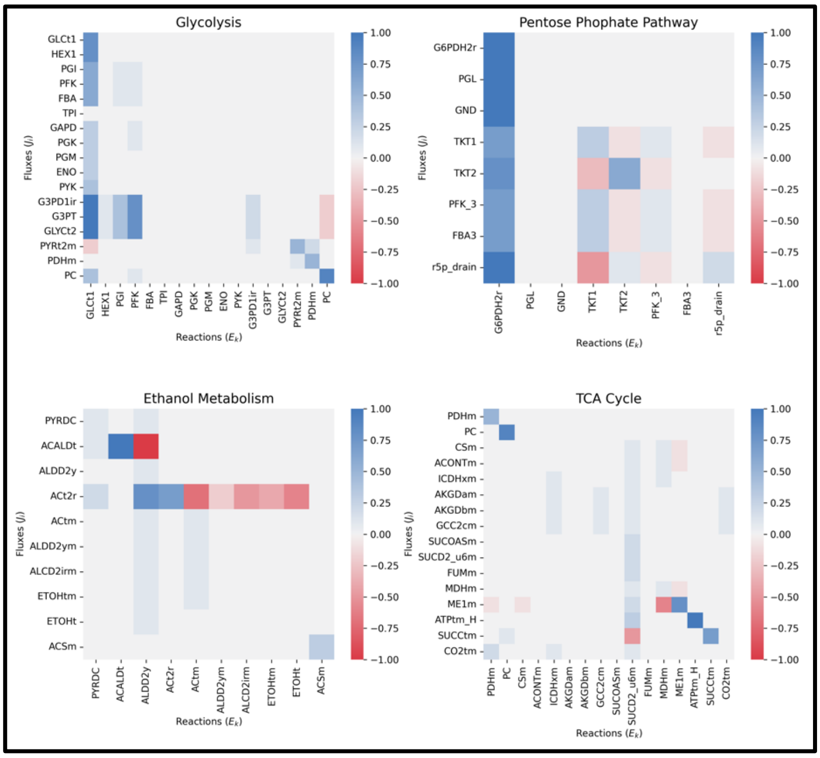

Figure S8: Metabolic control analysis heat-maps for cells growing in a phosphate-limited medium with a dilution rate of 0.11 1/hr, 100 sampled models. Positive values of C_E^J are indicated in blue, negative values are indicated in red.

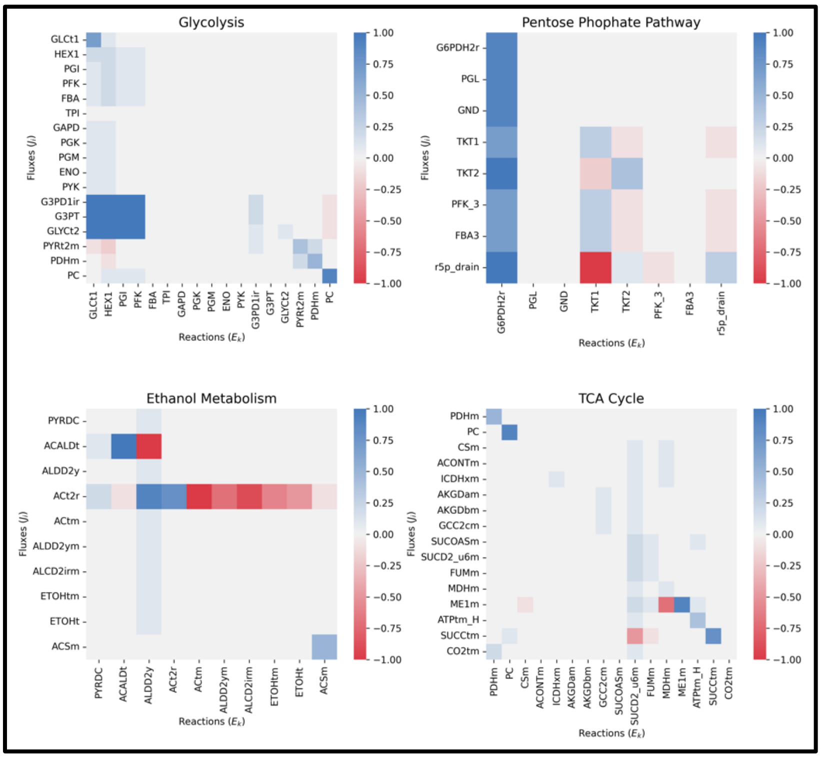

Figure S9: Metabolic control analysis heat-maps for cells growing in a nitrogen-limited medium with a dilution rate of 0.11 1/hr, 100 sampled models. Positive values of C_E^J are indicated in blue, negative values are indicated in red.

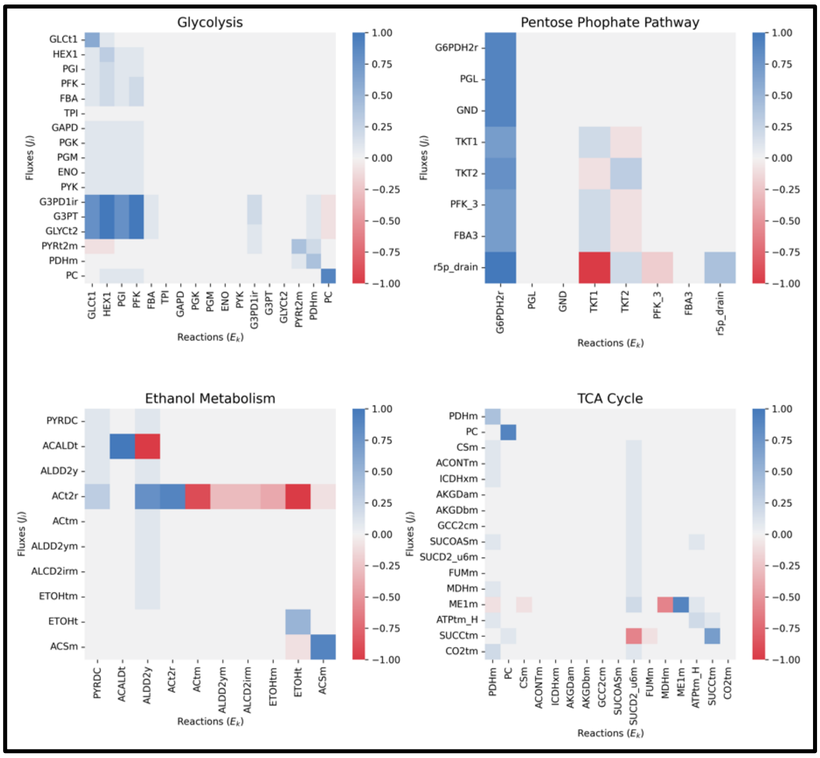

Figure S10: Metabolic control analysis heat-maps for cells growing in an uracil-limited medium with a dilution rate of 0.11 1/hr, 100 sampled models. Positive values of C_E^J are indicated in blue, negative values are indicated in red.

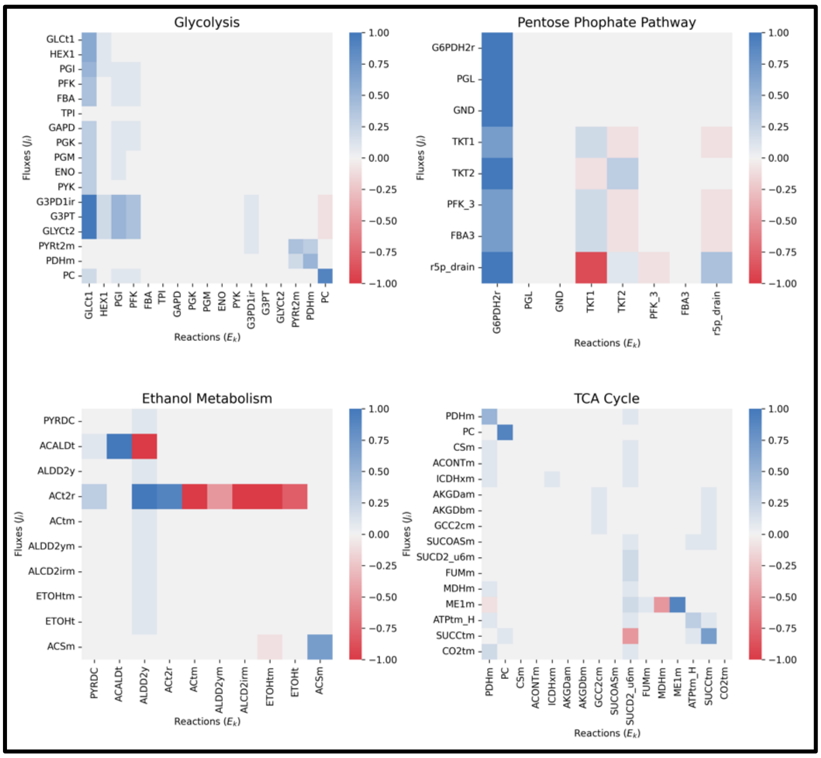

Figure S11: Metabolic control analysis heat-maps for cells growing in a leucine-limited medium with a dilution rate of 0.05 1/hr, 100 sampled models. Positive values of C_E^J are indicated in blue, negative values are indicated in red.

The Effect of Sample Size on the Performance of GRASP

Plots S12-S14 were produced with seaborn (Waskom 2021) and Matplotlib (Hunter 2007).

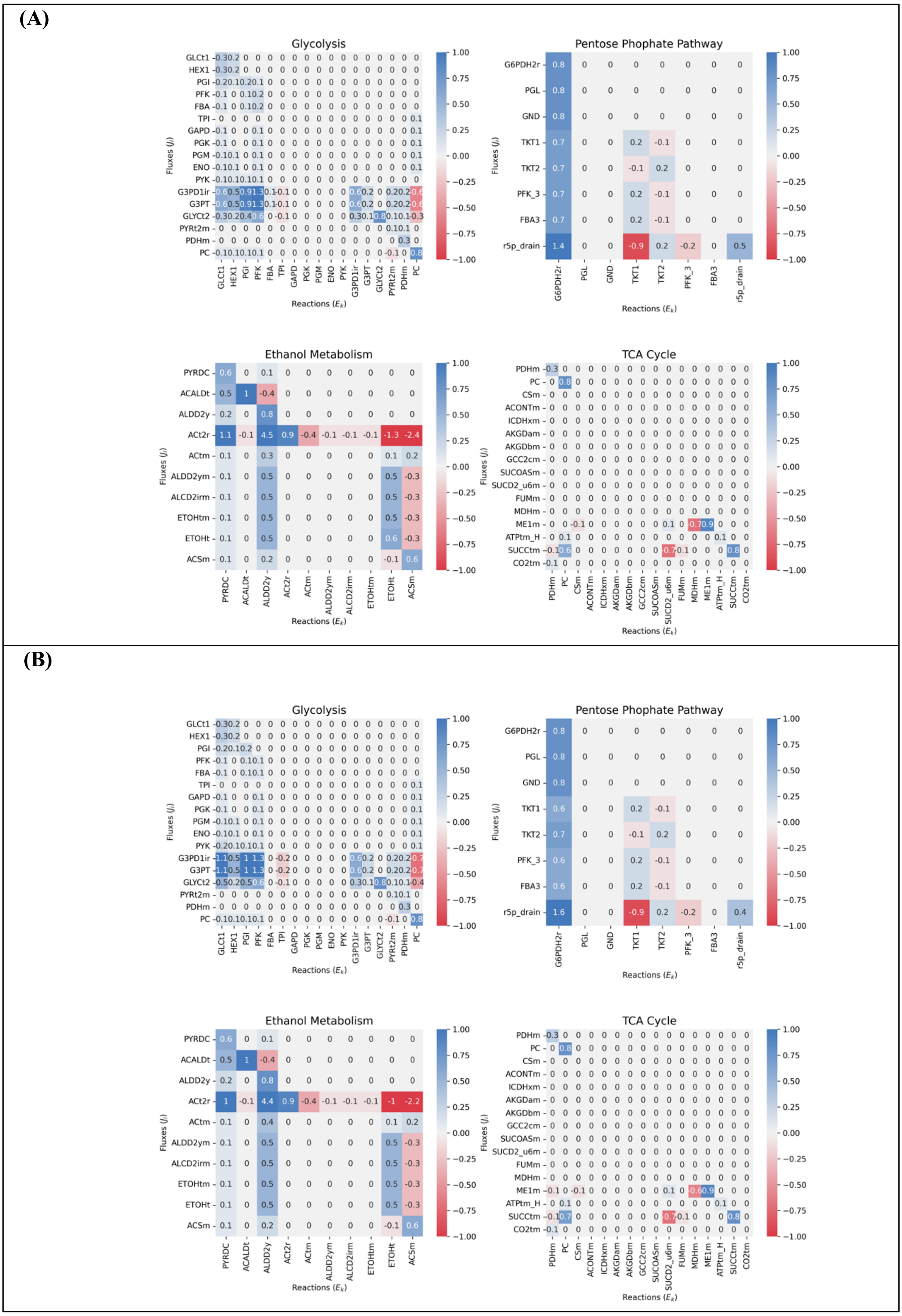

Figure S12: Metabolic control analysis heat-maps for cells growing in a glucose-limited medium with a dilution rate of 0.16 1/hr. Positive values of C_E^J are indicated in blue, negative values are indicated in red. A) Median values for 100 sampled models, B) Median values for 1000 sampled models.

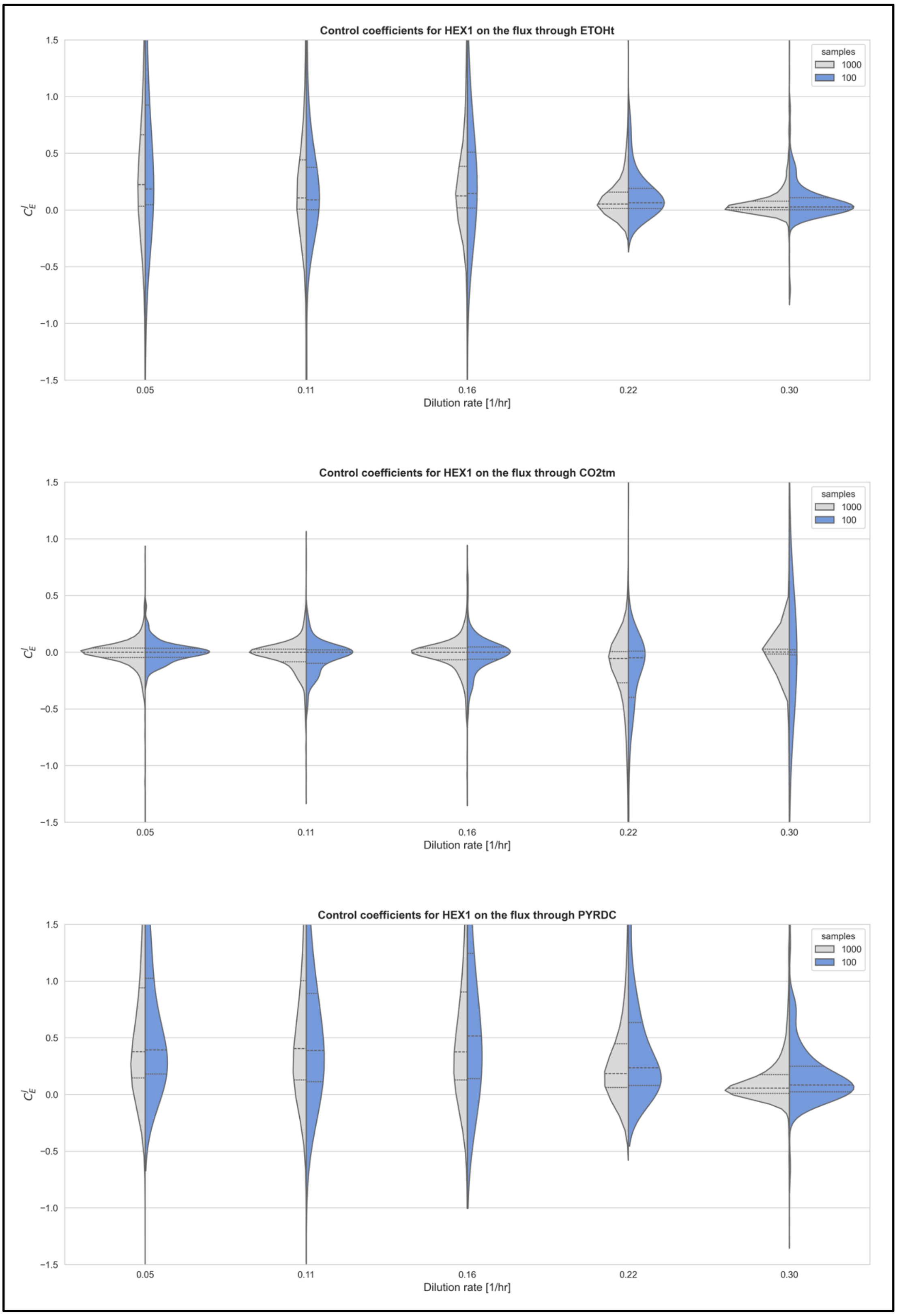

Figure S13: Distribution of the metabolic control coefficients for HEX1 on ethanol transport, carbon dioxide transport, and on the flux through PYRDC for cells growing in five different dilution rates of a glucose-limited medium. Blue: The first 100 sampled models out of a 1000, Grey: All 1000 sampled models.

Kolmogorov-Smirnov (K-S) test

We conducted a Kolmogorov-Smirnov test (Massey et al. 1951) to assess whether the distribution of control coefficients differs for each enzyme-flux pair when comparing 50, 75, 125 and 1000 sampled models to a baseline of 100 sampled models. For most pairs, the p-value was above 0.05, failing to reject the null hypothesis that the distributions belong to different populations. The tests were conducted using SciPy (Virtanen et al. 2020). Figure S14A presents the K-S test p-values for cells grown in a glucose-limited medium at a dilution rate of 0.22 hr^{-1}.

Out of the 6,724 enzyme-flux pairs, only a few showed p-values below 0.05, indicating significant differences in distribution. Specifically, when comparing 50 to 100 sampled models, 112 pairs had p-values below 0.05. This number dropped to zero when comparing 75 and 125 sampled models to the baseline. When comparing 1000 to 100 sampled models, 28 pairs (approximately 0.42%) showed significant differences. This result is not unexpected, as the K-S test is sensitive to sample size. When applying the Benjamini-Hochberg False Discovery Rate correction (Benjamini & Hochberg, 1995), none of the 6,724 corrected p-values was found to be below 0.05 (FDR level Q: 0.05). We therefore conclude that an ensemble of 100 sampled models is sufficient.

Median metabolic control coefficients vs. number of sampled models

In Figure S14(B), the median control coefficients for PYRDC across several fluxes are presented. As shown, the median values exhibit only small changes as more models are sampled. Moreover, we did not observe any meaningful shifts—such as substantial increases or decreases in the median values, or changes in their sign.

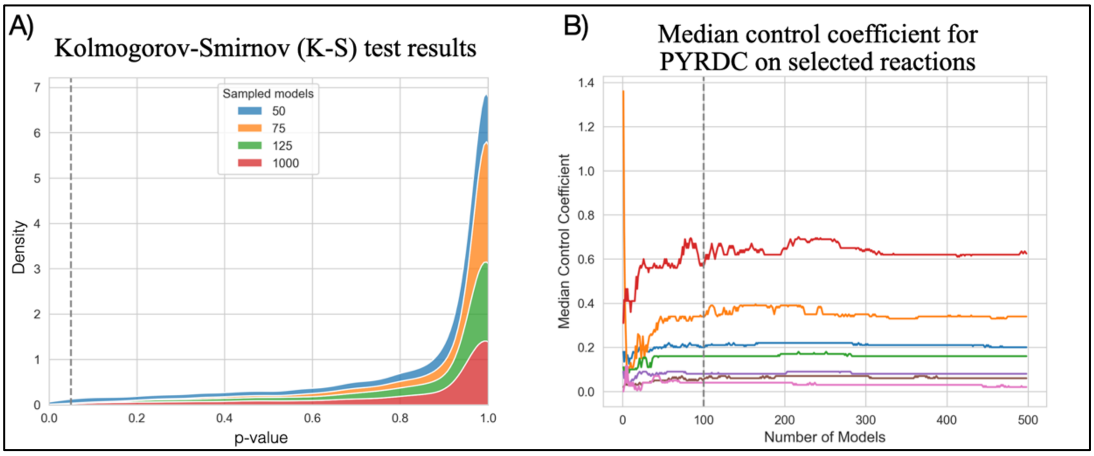

Figure S14: (A) Kernel Density Estimation Plot showing the K-S test results (p-values) for comparison of metabolic control coefficient distributions between ensembles of 50, 75, 125 and 1000 models versus 100 models. In total 6,724 enzyme-flux pairs were analyzed. Data shown for cells growing in a glucose-limited medium (dilution rate of 0.22 hr^{-1}). (B) Median metabolic control coefficients for selected PYRDC-flux pairs (Red: C_{PYRDC}^{ACt2r}, Orange: C_{PYRDC}^{ACALDt} , Blue: C_{PYRDC}^{PYRDC} , Green: C_{PYRDC}^{ALDD2y} , Purple: C_{PYRDC}^{ACtm} , Brown: C_{PYRDC}^{ETOHt}, Pink: C_{PYRDC}^{ACSm}). Data shown for cells growing in a glucose-limited medium (dilution rate of 0.22 hr^{-1}).

Figure S15 shows the relative change in control coefficient values versus the number of sampled models. The median control coefficients were calculated for each enzyme-flux pair every 10 sampled models. Hence, the first median was calculated for the first 10 sampled models, and the 2nd median was calculated for the first 20 sampled models and so on. Then, the relative change in median control coefficient was calculated and plotted against the number of sampled models. As can be seen, the relative change in the values of the median control coefficients decreases as we sample more models.

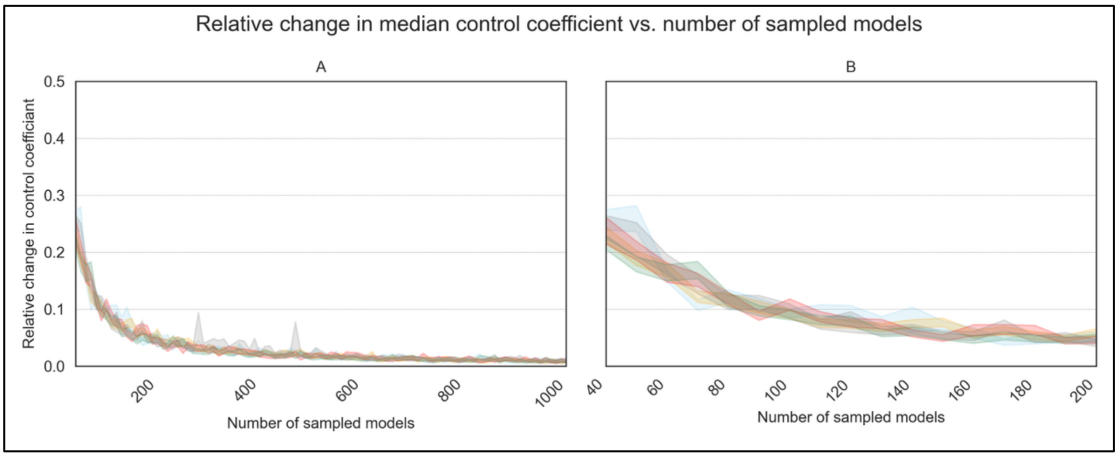

Figure S15: Relative changes in median control coefficients vs. number of sampled modes for the five glucose-limited growth conditions (grey: dilution rate of 0.30 1/hr; blue: dilution rate of 0.22 1/hr; red: dilution rate of 0.16 1/hr; orange: dilution rate of 0.11 1/hr; and green: dilution rate of 0.05 1/hr). (A) Ten to 1000 sampled models, (B) Ten to 200 sampled models. The shaded areas mark 95% confidence intervals. The plots were made with seaborn (Waskom 2021) and Matplotlib (Hunter 2007).

To further examine the convergence of the median control coefficients we decided to normalize them by dividing the median control coefficients calculated for each enzyme-flux pair after sampling further 10 models, by their final median control coefficient (calculated for 1000 sampled models). As can be seen, in Figure S16, while there are still small changes in median control coefficient after reaching 100 sampled models, these changes are minor and will most likely not influence the design strategy of metabolic engineers using the framework.

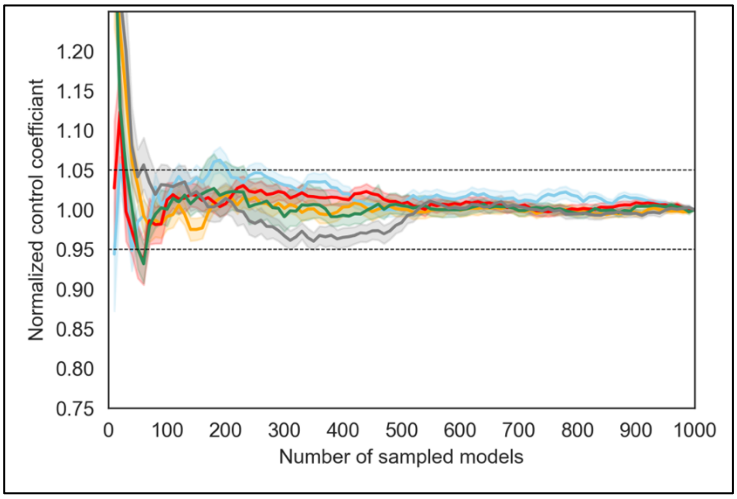

Figure S16: Normalized median control coefficients vs. number of sampled models for the five glucose-limited growth conditions (grey: dilution rate of 0.30 1/hr; blue: dilution rate of 0.22 1/hr; red: dilution rate of 0.16 1/hr; orange: dilution rate of 0.11 1/hr; and green: dilution rate of 0.05 1/hr).The shaded areas mark 95% confidence intervals and the solid line marks mean values. The plot was made with seaborn (Waskom 2021) and Matplotlib (Hunter 2007).

Allosteric effectors in our model

Allosteric effectors are molecules that bind to a regulatory site on an enzyme (not its active site) and influence the reaction rate. Four enzymes with allosteric effectors are included in our model: PFK, PYK, G3PD1 and ICDHxm (Isocitrate dehydrogenase NAD). In Figure S17 we show how the removal of the negative allosteric effect of ATP on PFK changes the control of PFK and HEX1 on glycerol secretion.

When the glucose concentration in the media is high, ATP has an important role in decreasing the activity of PFK (Larsson et al. 2000). The removal of ATP as an allosteric effector allows PFK to convert D-Fructose 6-phosphate into D-Fructose 1,6-bisphosphate without this important regulation. Thus, the control PFK has on glycolysis increases while the control of HEX1 decreases. In the case of a low glucose concentration in the media, the same removal of ATP as an allosteric effector will again lead to an increase in the control of PFK but will not decrease the control of HEX1, probably due to the lack of accumulation of glucose-6-phosphate.

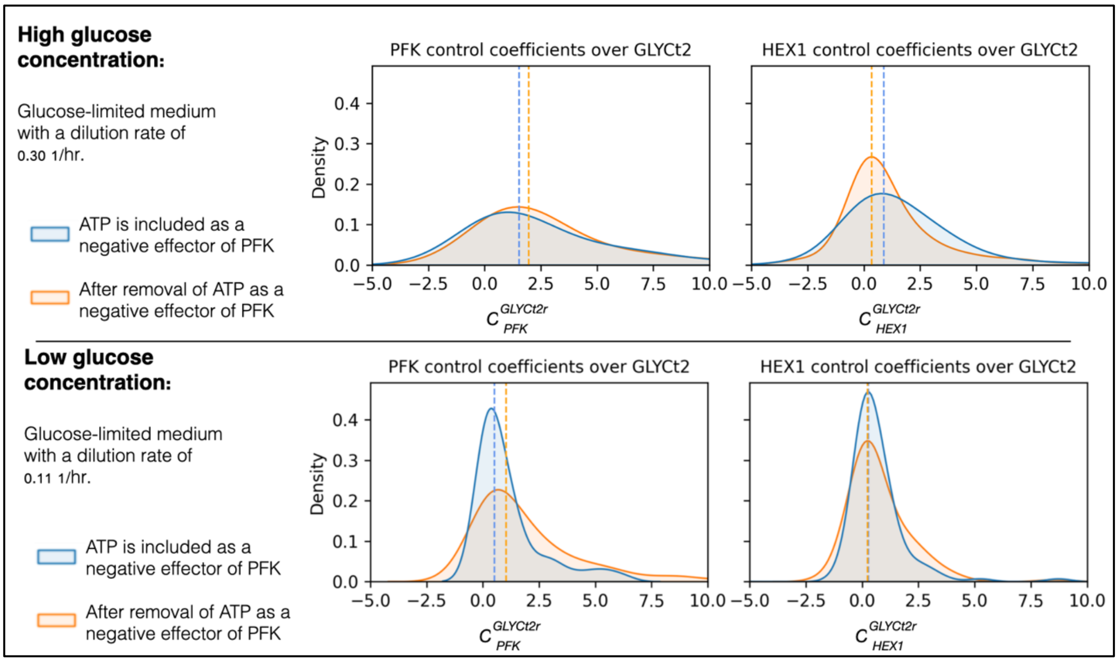

Figure S17: Kernel Density Estimation Plot showing the influence of ATP as an allosteric effector of PFK on the control of PFK and HEX1 over the secretion of glycerol. In this figure two examples are given, one when there is high concentration of glucose in the media and another when the concentration is low. The plot was made with seaborn (Waskom 2021) and Matplotlib (Hunter 2007).

References

1. Benjamini, Y., & Hochberg, Y. (1995). Controlling the false discovery rate: a practical and powerful approach to multiple testing. *Journal of the Royal statistical society: series B (Methodological)*, *57*(1), 289-300.
2. Bostian, K. A., & Betts, G. F. (1978). Kinetics and reaction mechanism of potassium-activated aldehyde dehydrogenase from Saccharomyces cerevisiae. *Biochemical Journal*, *173*(3), 787-798.
3. Cai, J., Pietzsch, M., Theobald, U., & Rizzi, M. (1996). Fast purification and kinetic studies of the glycerol-3-phosphate dehydrogenase from the yeast Saccharomyces cerevisiae. *Journal of biotechnology*, *49*(1-3), 19-27.
4. Gao, H., & Leary, J. A. (2003). Multiplex inhibitor screening and kinetic constant determinations for yeast hexokinase using mass spectrometry based assays. *Journal of the American Society for Mass Spectrometry*, *14*(3), 173-181.
5. He, W., Wang, Y., Liu, W., & Zhou, C. Z. (2007). Crystal structure of Saccharomyces cerevisiae 6-phosphogluconate dehydrogenase Gnd1. *BMC Structural Biology*, *7*, 1-9.
6. Hunter, J. D. (2007). Matplotlib: A 2D graphics environment. *Computing in science & engineering*, 9(03), 90-95.
7. Hynne, F., Danø, S., & Sørensen, P. G. (2001). Full-scale model of glycolysis in Saccharomyces cerevisiae. *Biophysical chemistry*, *94*(1-2), 121-163.
8. Jurica, M. S., Mesecar, A., Heath, P. J., Shi, W., Nowak, T., & Stoddard, B. L. (1998). The allosteric regulation of pyruvate kinase by fructose-1, 6-bisphosphate. *Structure*, *6*(2), 195-210.
9. King, Z. A., Dräger, A., Ebrahim, A., Sonnenschein, N., Lewis, N. E., & Palsson, B. O. (2015). Escher: a web application for building, sharing, and embedding data-rich visualizations of biological pathways. PLoS computational biology, 11(8), e1004321.
10. King, Z. A., Lu, J., Dräger, A., Miller, P., Federowicz, S., Lerman, J. A., ... & Lewis, N. E. (2016). BiGG Models: A platform for integrating, standardizing and sharing genome-scale models. *Nucleic acids research*, *44*(D1), D515-D522.
11. Larsson, C., Påhlman, I. L., & Gustafsson, L. (2000). The importance of ATP as a regulator of glycolytic flux in Saccharomyces cerevisiae. *Yeast*, *16*(9), 797-809.
12. Lin, A. P., & McAlister-Henn, L. (2003). Homologous binding sites in yeast isocitrate dehydrogenase for cofactor (NAD+) and allosteric activator (AMP). *Journal of Biological Chemistry*, *278*(15), 12864-12872.
13. Massey Jr, F. J. (1951). The Kolmogorov-Smirnov test for goodness of fit. *Journal of the American statistical Association*, 46(253), 68-78
14. McAlister, L., & Holland, M. J. (1985). Isolation and characterization of yeast strains carrying mutations in the glyceraldehyde-3-phosphate dehydrogenase genes. *Journal of Biological Chemistry*, *260*(28), 15013-15018.
15. Mulcahy, P., O'Flaherty, M., Jennings, L., & Griffin, T. (2002). Application of kinetic-based biospecific affinity chromatographic systems to ATP-dependent enzymes: studies with yeast hexokinase. *Analytical biochemistry*, *309*(2), 279-292.

16. Przybylski, Frank, Andreas Otto, Karl Nissler, Wolfgang Schellenberger, and Eberhard Hofmann. "Effects of fructose 1, 6-bisphosphate on the activation of yeast phosphofructokinase by fructose 2, 6-bisphosphate and AMP." Biochimica et Biophysica Acta (BBA)-Protein Structure and Molecular Enzymology 831, no. 3 (1985): 350-352.

17. Rojas-Pirela, M., Andrade-Alviárez, D., Rojas, V., Kemmerling, U., Cáceres, A. J., Michels, P. A., ... & Quiñones, W. (2020). Phosphoglycerate kinase: structural aspects and functions, with special emphasis on the enzyme from Kinetoplastea. *Open Biology*, *10*(11), 200302.

18. Schomburg, I., Chang, A., & Schomburg, D. (2002). BRENDA, enzyme data and metabolic information. *Nucleic acids research*, *30*(1), 47-49.

19. Schomburg, I., Jeske, L., Ulbrich, M., Placzek, S., Chang, A., & Schomburg, D. (2017). The BRENDA enzyme information system–From a database to an expert system. *Journal of biotechnology*, *261*, 194-206.

20. Sevostyanova, I. A., Selivanov, V. A., Yurshev, V. A., Solovjeva, O. N., Zabrodskaya, S. V., & Kochetov, G. A. (2009). Cooperative binding of substrates to transketolase from Saccharomyces cerevisiae. *Biochemistry (Moscow)*, *74*, 789-792.

21. Solovjeva, O. N., Selivanov, V. A., Orlov, V. N., & Kochetov, G. A. (2019). Stages of the formation of nonequivalence of active centers of transketolase from baker's yeast. *Molecular Catalysis*, *466*, 122-129.

22. UniProt Consortium, T. (2018). UniProt: the universal protein knowledgebase. *Nucleic acids research*, *46*(5), 2699-2699.

23. van Urk, H., Schipper, D., Breedveld, G. J., Mak, P. R., Scheffers, W. A., & van Dijken, J. P. (1989). Localization and kinetics of pyruvate-metabolizing enzymes in relation to aerobic alcoholic fermentation in Saccharomyces cerevisiae CBS 8066 and Candida utilis CBS 621. *Biochimica et Biophysica Acta (BBA)-General Subjects*, *992*(1), 78-86.

24. Virtanen, P., Gommers, R., Oliphant, T. E., Haberland, M., Reddy, T., Cournapeau, D., ... & Van Mulbregt, P. (2020). SciPy 1.0: fundamental algorithms for scientific computing in Python. *Nature methods*, *17*(3), 261-272.

25. Wang, Y., Wang, Q., Huang, H., Huang, W., Chen, Y., McGarvey, P. B., ... & UniProt Consortium. (2021). A crowdsourcing open platform for literature curation in UniProt. *PLoS biology*, *19*(12), e3001464.

26. Waskom, M. L. (2021). Seaborn: statistical data visualization. *Journal of open source software*, 6(60), 3021

27. Xu, Y. F., Zhao, X., Glass, D. S., Absalan, F., Perlman, D. H., Broach, J. R., & Rabinowitz, J. D. (2012). Regulation of yeast pyruvate kinase by ultrasensitive allostery independent of phosphorylation. *Molecular cell*, *48*(1), 52-62.
